# Spontaneous pregnancy after thread-embedding therapy treatment with premature ovarian insufficiency after unilateral oophorectomy: a case report

**DOI:** 10.3389/fmed.2024.1357824

**Published:** 2024-04-26

**Authors:** Ziniu Zhang, Jingxue Yuan, Jingjing Zhao, Yue Song, Jinxia Ni

**Affiliations:** Department of Acupuncture, Dongzhimen Hospital, Beijing University of Chinese Medicine, Beijing, China

**Keywords:** thread-embedding therapy, premature ovarian insufficiency, unilateral oophorectomy, pregnancy, acupuncture, case report

## Abstract

Premature ovarian insufficiency (POI) is a condition characterized by menstrual disturbance, subfertility, and estrogen deficiency symptoms. Women with POI have a small chance of natural conception, which may be even smaller when complicated with unilateral ovarian due to reduction of the ovarian follicular reserve. In China, acupuncture has been widely used to treat POI and POI-induced infertility, and studies have shown that acupuncture is helpful for improving ovarian function. Thread-embedding therapy is a method of acupuncture treatment development and extension, which can make the acupuncture effect last. In this article, we report a patient diagnosed with POI after unilateral oophorectomy (UO) who spontaneously conceived after thread-embedding therapy. Thread-embedding therapy may improve ovarian function in patients with POI, thereby providing a treatment strategy for infertility in patients with POI. This case report was written in accordance with the CARE guidelines.

## 1 Introduction

Premature ovarian insufficiency (POI) is a clinical syndrome defined by loss of ovarian activity that occurs in individuals less than 40 years old. It usually presents with menstrual disturbance (secondary amenorrhea or oligomenorrhea), subfertility, and estrogen deficiency symptoms (1, 2). The diagnostic criteria for POI are amenorrhea or oligomenorrhea (≥4 months) and two FSH levels higher than 25 IU/L (>4 weeks apart) according to the European Society of Human Reproduction and Embryology guidelines (1). The aetiologies of POI are still not fully determined, and its occurrence may be related to the following causes: chromosomal abnormalities, genetics, iatrogenic factors, autoimmunity, metabolism, infection, and environmental pollutants (3, 4). The global overall prevalence of POI was calculated as 3.7% (5). It has a significant negative impact on physical health, psychological well-being and the quality of life of patients (1, 6).

POI is one of the main factors affecting female fertility (7). Women with POI have a small chance of spontaneous pregnancy (1). Hormone replacement therapy is a main treatment for POI and can ameliorate the clinical complications caused by low estrogen levels (4). However, it has little or no benefit for improving the fertility rate and may increase the risk of ovarian cancer, breast cancer, endometrial cancer, thrombotic disease, meningioma, and other diseases (8, 9). Acupuncture therapy is a method of preventing and treating diseases that originated in ancient China. Due to its simple operation, satisfactory effect and low cost, acupuncture has been used increasingly frequently in clinical treatment (10). Thread-embedding therapy, as a type of acupuncture method, is widely used in the clinic. We will present a patient with POI after UO who conceived after thread-embedding therapy, which is rarely reported to our knowledge. The patient only retained her left ovary, which made pregnancy more difficult while experiencing POI. This study was approved by the ethics committee of Dongzhimen Hospital, Beijing University of Chinese Medicine (Approval no. 2022DZMEC-257–01). Signed written informed consents were obtained from the patient.

## 2 Case presentation

### 2.1 Medical history

A 35-year-old woman presented to Department of Acupuncture, Dongzhimen Hospital, Beijing University of Chinese Medicine complaining of oligomenorrhea for more than 4 months (weighed 60 kg and was 162 cm tall, body mass index [BMI] 23 kg/m^2^). She denied any thyroid (related blood tests listed in Table 1) and adrenal cortex function-related medical history, any related family history or any exposure to radioactive materials or anticancer drugs. The patient had regular cycles and a relatively large blood flow after menarche at 12 years of age, accompanied by dysmenorrhea and blood clots. She married at the age of 23 with no contraception all the time. In 2017, she received right oophorectomy due to the torsion of a right ovarian cyst. She reported that she began experiencing menstrual disorder after that operation. Unfortunately, relevant records and evidence were not available, and she did not receive any treatment. She conceived in the same year, but an inevitable abortion occurred in March 2018 because of a low lying placenta and preterm premature rupture of membranes. She took traditional Chinese medicine herbs intermittently for 6 months after the abortion, and the symptoms seemed to be relieved.

**Table 1:** Blood tests of thyroid function.

Item	Abbreviation	Result	Unit	Normal reference range
Antithyroid Peroxidase Antibody	aTPO	57.00^*^	U/ml	0–60
		<28.00^△^		
Free Thyroxine 4	FT4	16.20^*^	pmol/L	11.5–22.7
		15.36^△^		
Thyroid-stimulating Hormone	TSH3UL	1.18^*^	mIU/L	0.55–4.78
		3.71^△^		

^*^Tested on 22/02/2022. ^△^Tested on 02/04/2022.

However, the effect did not last long. She had not conceived for 2 years, so she tested her hormonal blood on August 4, 2020, which showed a high level of follicle-stimulating hormone (FSH) at 25.80 IU/L (normal reference range: follicular phase 3.85–8.78 IU/L), together with luteinizing hormone (LH) at 19.81 IU/L (normal reference range: follicular phase 2.12–10.89 IU/L) and estradiol (E2) at 78 pg./mL (normal reference range: follicular phase 27–122 pg./mL). A repeat test on September 12, 2020, at the same hospital confirmed POI (FSH: 37.80 IU/L, LH: 15.34 IU/L, E2: 25 pg./mL). Transvaginal sonography conducted on August 28, 2020 showed that the left ovary was 3.3 cm*1.8 cm in length and width, and the antral follicle count (AFC) was only 1. The left ovarian arterial blood flow was so low that the peak systolic velocity (PSV), end-diastolic velocity (EDV), resistance index (RI) and pulsatility index (PI) were all undetectable by Doppler blood flow measurements.

POI symptoms also exist. In August 2020, her modified Kupperman index (mKMI) (11) was 24, with moderate symptoms of insomnia, depression and suspicion, vertigo, fatigue, arthralgia and myalgia as well as mild symptoms of vasomotor, paraesthesia, nervousness, urinary symptoms and dyspareunia. Her score of TCM syndrome [developed referring to Guidance Principle of Clinical Study on New Drug of Traditional Chinese Medicine (12) and Gynaecology of Traditional Chinese Medicine (13)] was 22 with the following symptoms: menses occurred at least once every 4 months and lasted 1–2 days, and the period was delayed for 1–2 weeks; menstrual blood had a 2/3 reduction in volume from the original, which was slightly thick and dark red in colour; moderate symptoms of waist and knee soreness, hyposexuality, vaginal dryness, dizziness, tinnitus, dry mouth, poor sleep quality, dreaming and irritability; and a mild symptom of hot flashes and sweating. Physical examination was normal.

### 2.2 Treatment process

**Figure 1:**
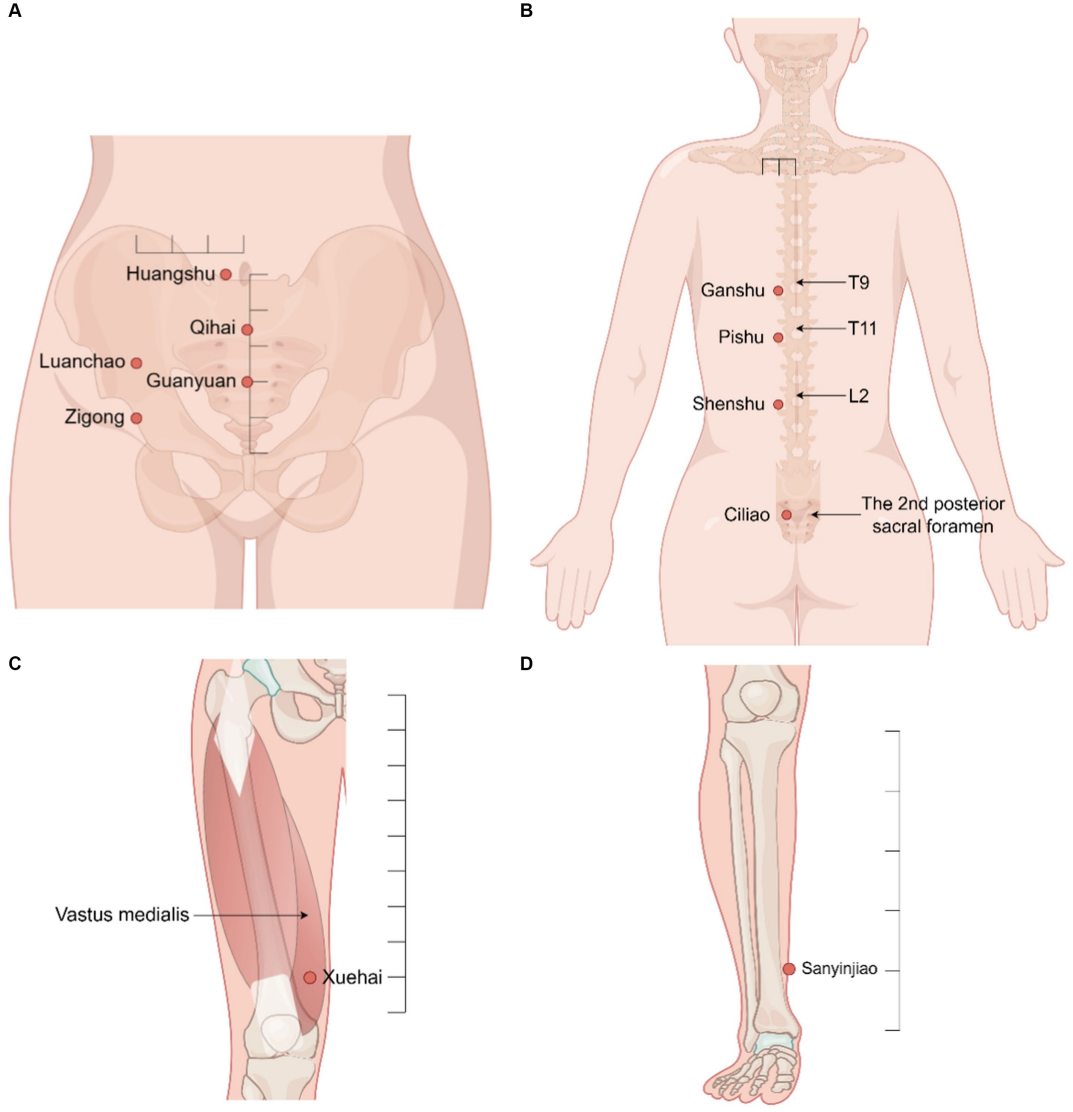
Location of acupoints on the human body. By Figdraw. **(A)** Abdominal acupoints. **(B)** Dorsal acupoints. **(C)** Femoral acupoints. **(D)** Crural acupoints.

**Figure 2:**
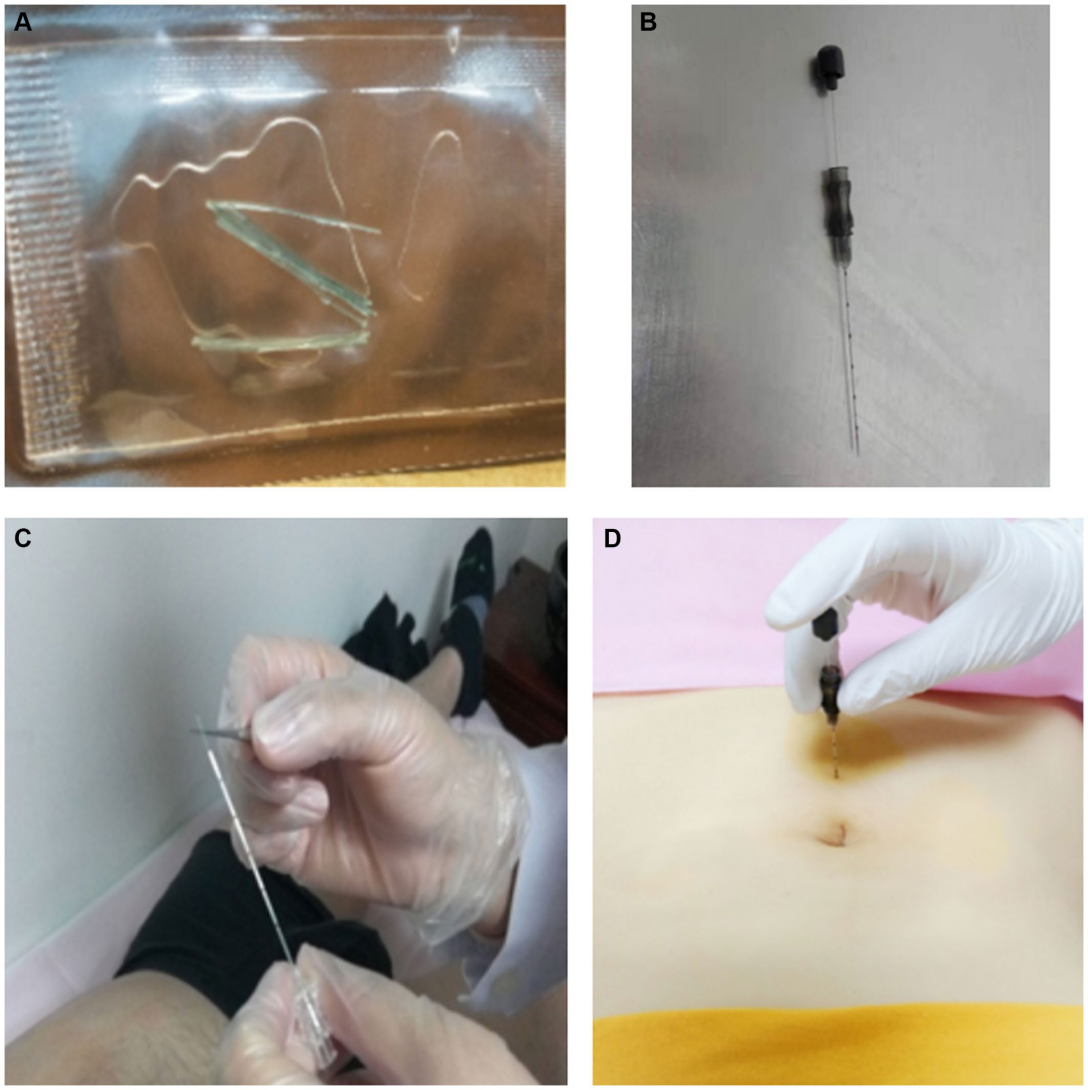
Process of thread-embedding therapy. **(A)** Absorbable surgical suture. **(B)** Disposable thread-embedding needle. **(C)** Place the thread in the front of the tube of the needle. **(D)** Puncture the needle into the acupoint. Then, push the needle core while withdrawing the needle tube at the same time.

We prescribed thread-embedding therapy once every 2 weeks for the patient. The following acupoints were chosen: Huangshu (KI16 bilateral), Qihai (CV6), Guanyuan (CV4), Zigong (EX-CA1 bilateral), Luanchao (bilateral), Xuehai (SP10 bilateral), Sanyinjiao (SP6 bilateral), Ganshu (BL18 bilateral), Pishu (BL20 bilateral), Shenshu (BL23 bilateral), and Ciliao (BL32 bilateral). Their location are based on the National Standards of P. R GB∕T 12346–2021 Nomenclature and location of meridian points and National Standards of P. R GB∕T 40997–2021 nomenclature and location of extra points in common use. Figure 1 illustrates their location on the human body. Through the thread-embedding needle, a short segment of absorbable collagen thread will be embedded into acupoints, together with the adjustment of the needling angle, to evoke a “de qi” sensation (manifesting as soreness, numbness, heaviness, and distention). The operating method follows the National Standards of P.R GB/T 21709.10–2008 Standard manipulations of acupuncture and moxibustion—Part 10 Thread-embedding therapy. In short, the patient were placed in the supine position first and then in the prone position, and the acpoints was fully exposed and disinfected. The operator cleaned and disinfected his or her hands, opened the embedding bag, wore sterile gloves, and took a 4–0 absorbable surgical suture with a length of 2 cm (Shandong Boda Medical Products CO., LTD, BD150301, Figure 2A) with sterile tweezers. It was placed in the front of the tube of a 0.7 mm × 60 mm disposable thread-embedding needle (Zhenjiang Gaoguan Medical Equipment Co., LTD, YZB/Su 0480–2007, Figures 2B,C), and the thread was loaded into the needle body from the tip (the needle core was slightly back so that the thread was flush with the inner edge of the needle tip). The angles and depths of needling at each acupuncture point are listed in Table 2, which is appropriate to feel acid and swelling at the acupoints specifically. Then, push the needle core while withdrawing the needle tube at the same time (Figure 2D). After the needle is out, press the acupoint with a sterilized cotton swab for a moment to prevent bleeding. The patient described her experience of thread-embedding therapy as “feeling good physically.”

**Table 2:** The angle and depth of needle insertion at each point.

Acupoint	Angle	Depth
Huangshu (KI16, bilateral)	45 degree downward slant with the skin.	2.5–3 cm
Guanyuan (CV4)	45 degree downward slant with the skin.	2.5–3 cm
Qihai (CV6)	45 degree downward slant with the skin.	2.5–3 cm
Zigong (EX-CA1, bilateral)	Oblique stab at 45 degrees to the skin, toward the pubic symphysis.	2.5–3 cm
Luanchao* (bilateral)	Oblique stab at 45 degrees to the skin, toward the pubic symphysis.	2.5–3 cm
Sanyinjiao (SP6, bilateral)	45 degree downward slant with the skin	2.5–3 cm
Xuehai (SP10 bilateral)	45 degree downward slant with the skin	3–3.5 cm
Ganshu (BL18, bilateral)	45 degree downward slant with the skin	2.5–3 cm
Pishu (BL20, bilateral)	45 degree downward slant with the skin	2.5–3 cm
Shenshu (BL23, bilateral)	perpendicular	2.5–3 cm
Ciliao (BL32, bilateral)	Stabbing into the second posterior sacral foramen.	4–5 cm

^*^ The acupoint Luanchao has not yet been listed in the National Standard. It was developed according to the anatomical location of the ovary by modern doctors and is effective both in experience and clinical trials, but the specific positioning is controversial (14–16). In this study, the longitudinal coordinates of the acupoint Luanchao are located at the midpoint between the umbilicus and the pubic symphysis, and the horizontal coordinates are located at the intersection between the umbilicus and the outer 3/4 of the nipple.

### 2.3 Follow up and outcomes

**Figure 3:**
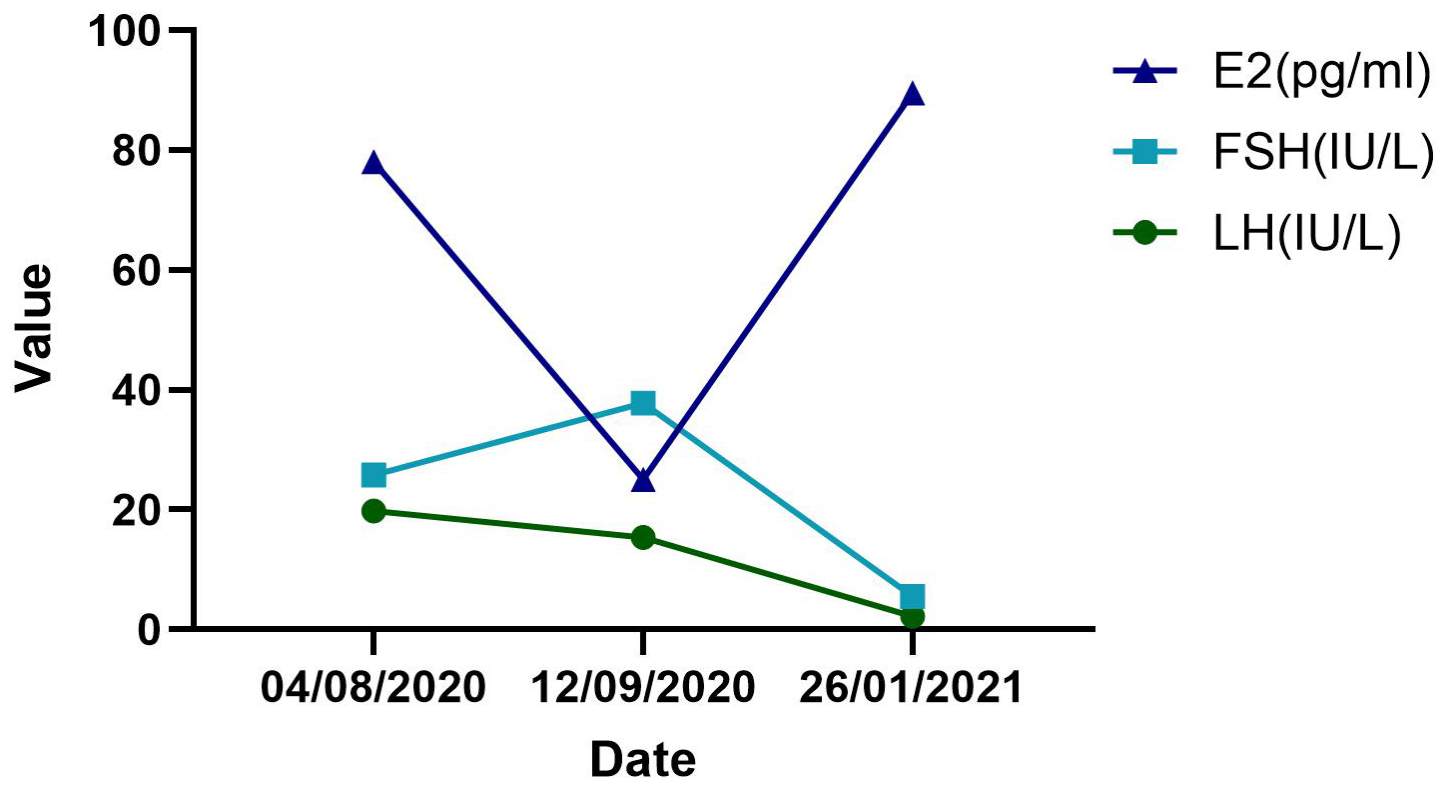
Female hormonal change values. August 4, 2020 and September 12, 2020 were the times before treatment. January 26, 2021 was the time during the treatment period.

**Figure 4:**
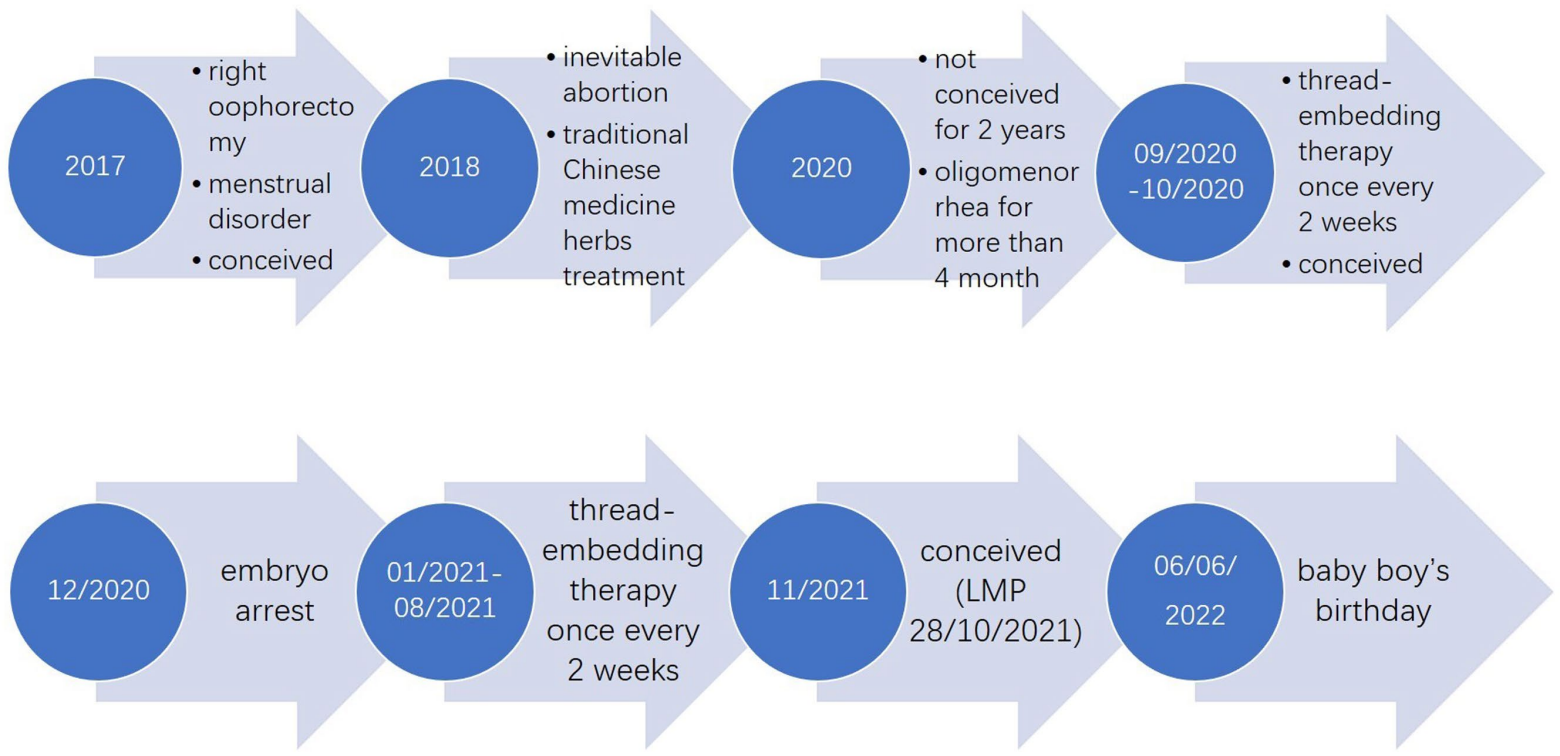
Treatment timeline. Contents in the arrow lists the main events in treatment and the development and outcome of the disease at the time labelled in the circle at the bottom of the arrow.

After 4 rounds of thread-embedding, the patient conceived in October 2020 and thus suspended the therapy. At that time, her mKMI was reduced sharply to 8, and the score of TCM syndrome was reduced to 11. Unfortunately, curettage was performed in December 2020 because of embryo arrest. One month later, she continued to receive the therapy once every 2 weeks for 8 months, which was sustained until August 2021. The follow-up in January 2021 showed an even better outcome in mKMI and TCM syndrome scores of 0 and 8, respectively. A hormonal blood test conducted on January 26, 2021, also showed that all three indices had returned to normal (FSH: 5.53 IU/L, LH: 2.24 IU/L, E2: 89.53 pg./mL). Each of the patient’s sex hormones was measured during the second to the fourth day of menstruation. The details of her mKMI and score of TCM syndrome are shown in Tables 3, 4. The changes in her hormone level and the treatment timeline are demonstrated, respectively, in Figures 3, 4.

**Table 3:** mKMI*.

Symptoms	Weighting score	Grades				Score		
		0	1	2	3	August 2020	October 2020	January 2021
Vasomotor	4	Never	Less than 3 times per day	3–9 times per day	More than 10 times per day	1	0	0
Paraesthesia	2	Never	Some-times	Often tingling numbness, tinnitus, etc.	Often and severe	1	0	0
Insomnia	2	Never	Some-times	Often	Often and severe, need medication	2	1	0
Nervousness	2	Never	Some-times	Often	Often and uncontrollable	1	0	0
Urinary symptoms	2	Never	Some-times	Controllable	Uncontrollable	1	0	0
Dyspareunia	2	Never	Some-times	Controllable	Unendurable	1	1	0
Depression and suspicion	1	Never	Some-times	Often but controllable	Lose faith in life	2	1	0
Vertigo	1	Never	Some-times	Often but have no impact on life	Have impact on life and work	2	1	0
Fatigue	1	Never	Some-times	Often	Restrictions on daily life	2	1	0
Arthralgia and myalgia	1	Never	Some-times	Often but functional normal	dysfunction	2	1	0
Headaches	1	Never	Some-times	Often but endurable	Often and need medication	0	0	0
Palpitation	1	Never	Some-times	Often but have no impact on work	Need to be treated	0	0	0
Formication	1	Never	Some-times	Often but endurable	Need to be treated	0	0	0
Total	24						8	0

^*^The symptom score is the product of the score and the weighting score, and the total score is the sum of the symptom scores. The higher the total score, the more pronounced the menopausal symptoms.

**Table 4:** TCM syndrome scores*.

Symptoms	Grades				Score		
	0	1	2	3	August 2020	October 2020	January 2021
Oligomenorrhea	Normal	At least once every 4 months	Once every 4–6 months	Amenorrhea	1	0	0
Delayed menses	Normal	For 1–2 weeks	For 2–3 weeks	For more than 3 weeks	1	0	0
Menstrual period	More than 5 days	3–4 days	1–2 days	None	2	1	1
Menstrual blood volume	Normal	Reduce to 1/2	Reduce to 1/3	Few drops	2	1	1
Colour and thickness	Normal	Dark red and slightly thick	Red–purple and rather thick	Dark purple and very thick	1	1	1
Waist and knee soreness	Never	Sometimes	Often but have no impact on life	Often and need to be treated	2	1	0
Hyposexuality	Never	Decreased sexual desire	Little sexual desire	No sexual desire	2	1	0
Vaginal dryness	Never	Sometimes	Have no impact on sexual life	Have impact on sexual life	2	1	1
Dizziness and tinnitus	Never	Sometimes	Often but have no impact on life	Often and have impact on life	2	2	1
Dry mouth	Never	Slightly	Can be relieved by drinking water	Desire water but have no relief	2	1	1
Hot flashes and sweating	Never	Less than 3 times per day	3–9 times per day	More than 10 times per day	1	0	0
Poor sleep quality and dreaming	Never	Dream a lot, sleep less than 4–5 h per night	Sleep less than 2–3 h per night and have difficulty falling asleep	Pernoctation	2	1	1
Irritability	Never	Sometimes	Often but can keep the normal life	Last all day and have Impact on work	2	1	1
Total					22	11	8

^*^The patient’s score corresponds to the severity of the symptoms, with no symptoms being 0, mild being 1, moderate being 2, and severe being 3. The total score is the sum of each symptom score.

In November 2021, the patient celebrated her third pregnancy. Her last menstrual period (LMP) was October 28, 2021, and she gave birth to a baby boy by spontaneous labour on June 6, 2022. The baby is in good health.

## 3 Discussion

As a patient with only one ovary, of which arterial blood flow was too low to detect, the function of her ovary was low. The increase in FSH and dramatic decrease in E2 between August 4, 2020, and September 12, 2020, may also suggest that her ovarian function was declining sharply at the beginning, adding challenges to our treatment. In this circumstance, the two pregnancies after treatment were truly inspiring, and the three main serum sex hormones we tested (FSH, LH, and E2, January 26, 2021) had all returned to normal. The former conception did not turn out well, indicating that management in pregnancy still requires attention among patients with POI. Second, the significant decreases in mKMI and TCM syndrome scores were notable. The menopausal symptoms disappeared completely, and her menstrual cycle was regular. She claimed to take proprietary Chinese medicine such as Baotai Cuyun Pill and Xianlingpi capsule for a short period of time during her thread-embedding therapy, but we tend to consider it only a supporting role due to the small dosage and short duration. In addition, the patient did not receive any treatment other than four thread-embedding therapies between August and October 2020. At that time, she also conceived and showed progress for mKMI and TCM syndrome scores, indicating promotion of ovarian function and an independent therapeutic effect of thread-embedding therapy.

POI is a condition in which there is a decline in ovarian function in women who are younger than 40 years resulting in a hypo-oestrogenic state with elevated gonadotrophins and oligomenorrhoea/amenorrhoea. This leads to short term complications of menopausal symptoms and long-term effects on bone and cardiovascular health, cognition as well as the impact of reduced fertility and sexual function associated with this condition (17). A study reported that the prevalence of infertility is approximately 9%, with more than half of couples seeking medical care (18). POI is one of the main causes of infertility in women (1). The impact of UO to fertility is controversial. Some supposed that the removal of one ovary does not significantly reduce the pregnancy or fertility outcome respect to other abdominal or pelvic procedures (19, 20), while others showed a significant detrimental effect of UO on the rates of live birth and pregnancy (21), or have less IVF capacity but the same pregnancy rate (22). When excluded assisted reproductive technology, the spontaneous pregnancy after UO is rarely reported. Yet UO can still be considered as a limitation on account of the reduction of ovarian follicular reserve (23).

Acupuncture therapy is an external treatment of traditional Chinese medicine and has been applied for POI. A prospective consecutive case series study suggested that acupuncture may decrease serum FSH and LH levels, raise serum E2 levels, improve menopausal symptoms, and reduce mental stress in patients with no serious side effects (24). Similarly, in another prospective observational study, the authors noted that electroacupuncture may modulate reproductive hormone levels with no significant side effects (25). A meta-analysis of acupuncture for POI showed that acupuncture could decrease FSH levels and improve menstruation (26). Modern medicine shows that the effectiveness of acupuncture in treating POI is mainly attributed to the improvement of ovarian function by regulating the neuroendocrine system and increasing blood supply to the reproductive organs (25, 27).

Thread-embedding therapy belongs to the range of acupuncture therapy. It is a kind of prevention and treatment technology that exerts long-lasting stimulatory effects on acupoints by incorporating absorbable surgical sutures into the acupoints (18). Thread-embedding therapy may combine the acupuncture effect, the absorption effect of the thread and the repair effect after the injury, integrating the needle and buried needle effect into one. It is the development of “needle retention” in traditional acupuncture by modern technology. Through the liquefaction and absorption process of absorbable protein lines, the acupoints are stimulated continuously and stably. *The Internal Classic* is the earliest classical work of TCM, and it says if the needle is not retained long enough then the persistent disease cannot be removed, which is the most significant difference and innovation from traditional acupuncture. In addition, it has the advantage of a lower treatment frequency, which can save time and improve the compliance among patients. This treatment method is widely used in our clinic and has advantages for POI. The adverse effects of thread-embedding therapy generally include subcutaneous bleeding, hard nodules, itching, redness, swelling, and heat pain, which may be related to the material of the thread and different personal physical conditions. The absorbable collagen threads used in this study had low antigenicity and good histocompatibility. In our report, the patient only experienced bleeding during treatment, which is a predictable event usually caused by a puncture on a vessel and can be stopped by applying pressure to the puncture site with a cotton swab. There were no other serious adverse events.

In this article, we report a patient with POI after UO who became pregnant spontaneously after thread-embedding therapy. We infer that thread-embedding therapy might be effective for POI in ameliorating serum sex hormone levels, improving ovarian function, and helping pregnancy in clinical treatment. The cause of POI is unknown in 85–90% of cases, and the proportion of different causes was also not determined overall (17, 28). In addition to the iatrogenic POI involved in this study, it can also be spontaneous. Based on the theory of traditional Chinese medicine, the mechanism of modern medicine and our clinical experience, we believe that other types of POI can also have a try to refer to this method. Possible mechanism of thread-embedding instead of traditional acupuncture in the treatment of POI still needs to be further explored.

One of the important factors influencing the clinical effectiveness of thread-embedding therapy is the selection of acupoints. In addition to the localized acupoints to be selected, some other acupoints were also vital and worthy of being chosen. In TCM theory, the kidney collects essence-qi and is closely related to female pregnancy. The liver is the master of drainage, which helps the kidney to collect and discharge essence-qi and influences the opening and closure of the uterus. The spleen is the foundation of the acquirement, which transports and transforms water and grain essence and can replenish the kidney essence. Furthermore, these three organs also have connections to the ovaries in meridians. Thus, the majority of selected acupoints in our treatment were located at the Liver Meridian of Foot-Jueyin, Kidney Meridian of Foot-Shaoyin, and Spleen Meridian of Foot-Taiyin. Additionally, the back-shu of the liver, kidney, and spleen were also indispensable.

There are also limitations to our report. The article was a retrospective report in which we gathered relevant information depending on the patient’s narrative. Patients may experience recall bias, which would not facilitate our collection and analysis of complete treatment information. In addition, based on the study design, the grade of evidence is low. Hence, the effectiveness and safety of thread-embedding therapy for POI need to be confirmed by more high-quality evidence.

## Data availability statement

The original contributions presented in the study are included in the article, further inquiries can be directed to the corresponding author/s.

## Ethics statement

The studies involving humans were approved by the Ethics Committee of Dongzhimen Hospital Affiliated to Beijing University of Chinese Medicine. The studies were conducted in accordance with the local legislation and institutional requirements. The participants provided their written informed consent to participate in this study. Written informed consent was obtained from the individual(s) for the publication of any potentially identifiable images or data included in this article.

## Author contributions

ZZ: Data curation, Writing – original draft. JY: Writing – original draft, Writing – review & editing. JZ: Data curation, Writing – original draft. YS: Investigation, Writing – original draft. JN: Project administration, Supervision, Writing – review & editing.
